# Cosmetic Preservatives: Hazardous Micropollutants in Need of Greater Attention?

**DOI:** 10.3390/ijms232214495

**Published:** 2022-11-21

**Authors:** Marta Nowak-Lange, Katarzyna Niedziałkowska, Katarzyna Lisowska

**Affiliations:** Department of Industrial Microbiology and Biotechnology, Faculty of Biology and Environmental Protection, University of Lodz, 12/16 Banacha Street, 90-237 Łódź, Poland

**Keywords:** preservatives, ecotoxicity, microbial degradation, personal care products, triclocarban, chloroxylenol, methylisothiazolinone, benzalkonium chloride

## Abstract

In recent years, personal care products (PCPs) have surfaced as a novel class of pollutants due to their release into wastewater treatment plants (WWTPs) and receiving environments by sewage effluent and biosolid-augmentation soil, which poses potential risks to non-target organisms. Among PCPs, there are preservatives that are added to cosmetics for protection against microbial spoilage. This paper presents a review of the occurrence in different environmental matrices, toxicological effects, and mechanisms of microbial degradation of four selected preservatives (triclocarban, chloroxylenol, methylisothiazolinone, and benzalkonium chloride). Due to the insufficient removal from WWTPs, cosmetic preservatives have been widely detected in aquatic environments and sewage sludge at concentrations mainly below tens of µg L^-1^. These compounds are toxic to aquatic organisms, such as fish, algae, daphnids, and rotifers, as well as terrestrial organisms. A summary of the mechanisms of preservative biodegradation by micro-organisms and analysis of emerging intermediates is also provided. Formed metabolites are often characterized by lower toxicity compared to the parent compounds. Further studies are needed for an evaluation of environmental concentrations of preservatives in diverse matrices and toxicity to more species of aquatic and terrestrial organisms, and for an understanding of the mechanisms of microbial degradation. The research should focus on chloroxylenol and methylisothiazolinone because these compounds are the least understood.

## 1. Introduction

The control and monitoring of environmental pollution have so far focused on priority pollutants that are regulated and considered hazardous, toxic, persistent, or accumulative. However, in the last decade, there has been a significant increase in interest in the occurrence of new pollutants and their fate in the environment and potential toxicity. Many of these substances are not newly discovered chemicals but compounds that have been used for decades and are only now questionable. Many of them are considered potentially significant environmental pollutants despite the lack of regulations and restrictions [1,2,3].

Among these pollutants, there are additives used in daily products named personal care products (PCPs), which are used to improve the quality of everyday life. The global beauty market (GMB) is usually divided into five main business sectors: hair care, skin care, color (makeup), fragrances, and toiletries. Statistical studies conducted in the United States have shown that one woman and one man use 12 and 6 cosmetics products per day, respectively [4]. The widespread use of PCPs, the cult of beauty, and strong competition in the cosmetics market make this industry one of the fastest growing industries in the world. The majority of global premium cosmetics sales is concentrated within the developed markets (mostly the USA, Japan, and France). For the production of cosmetics, slightly more than 12 thousand chemicals are used of which less than 20% have been recognized as completely safe for human health and the environment [5,6]. In addition, not all countries are required to control products entering the market. Therefore, in the last few years there have been concerns about the widespread use of personal care products and their impact on the environment [4]. These compounds are released into the environment, mainly from anthropogenic sources, and are defined by the Environmental Protection Agency of the U.S. (US EPA) as new compounds without regulatory status, whose impact on the environment and human health is poorly understood [7]. PCPs are most often intended for external use; thus, they directly reach sewage treatment plants and are not subject to previous metabolic changes. Their extensive use and improper disposal contribute to the contamination of soils and aquatic ecosystems by PCPs. The largest sources of PCPs are sewage effluents from wastewater treatment plants (WWTPs) [8,9], where the removal of PCPs from many is still unsatisfactory and requires a continuous optimization of the elimination processes. The treated effluents are discharged into receiving waters, including small streams, rivers, and lakes, and there are even places where the wastewater is released into the environment without previous treatment, being directly discharged into riverine habitats or water bodies. Reclaimed water and sewage sludge can be used in agricultural production and become a source of soil pollution. Excessive penetration of PCPs into the environment may contribute to the accumulation of these xenobiotics in the soil, where they can then enter groundwater or be absorbed by plants and crops and enter food chains. As they occur in the natural environment in low concentrations (ng L^−1^, µg L^−1^), they are known as micropollutants. Furthermore, due to their continuous entry into the environment and synergic effects resulting from the coupled parallel activity, even low concentrations of chemicals may have undesirable consequences in the environment [8,10,11,12,13,14,15,16,17,18,19,20,21,22].

Due to the global use of PCPs and their potential for negative effects in humans and wildlife, a rising number of studies have assessed the presence of additives for PCPs in environmental matrices. This review summarizes recent publications regarding the occurrence, toxicity, and transformation of most ecotoxic cosmetic preservatives found in the natural environment.

## 2. Characteristics of Preservatives in Cosmetics

The presence of water, a large amount of nutrients, and the way the consumer uses cosmetics promote the proliferation of micro-organisms in selected products. Such impurities can pose a threat to the health of the users and also adversely affect the organoleptic properties of the products [23,24]. In order to prevent microbial growth in cosmetics while extending their shelf life and the period of use of the products, most manufacturers use synthetic preservatives. Because of their biological activity, preservatives present a wide spectrum of undesirable effects for consumers, such as toxicity, irritation, or sensitization. Therefore, the safe use of these compounds is always being called into question. Nowadays, more and more producers decide not to use traditional preservatives in view of the negative public opinion about them. Manufactures who use new and little known chemicals claim that their products are “free” from potentially toxic compounds. Literature data of the possible harmful effects of some preservatives have led to increasing various international regulations and some chemicals have been banned in cosmetic products. In the European Union, the European Chemical Agency (ECHA) created a list of compounds for personal care product preservation from microbial spoilage, according to Annex V, Regulation 1223/2009/EC on Cosmetic Products, as amended by Regulation (EU) 2021/1902, 3 November 2021 [25]. In the United States, the Cosmetic Ingredient Review (CIR), led by a panel of medical experts, collaborates with the US Food and Drug Administration (FDA) to provide a review and assessment of the safety of ingredients used in cosmetics. The regulations regard the type and amount of preservatives added to cosmetics; nevertheless, the regulatory issues concerning preservatives in cosmetics are different in other countries [23,26,27]. It is important to point out that the regulatory status of preservatives is very dynamic and varies from region to region and even from country to country [27,28].

The microbial stability of cosmetic preparations without preservatives is very short; therefore, it is impossible to completely exclude them from cosmetic products manufactured on a large scale. There are some characteristics to take into consideration in preservative selection. The agents should have a broad spectrum and be active against all possible bacteria and fungi. When choosing preservatives, their stability is also very important in a wide range of pH values and temperatures, a lack of interaction with other cosmetic ingredients, and resistance to light and oxygen. Compounds serving as preservatives should be colorless, tasteless, and palpable and should not undergo hydrolysis. All of these features mean that these substances can be considered potentially harmful to the environment and to humans. Furthermore, given their widespread use in daily life, treatment of environmental contamination is challenging, as preservative avoidance may be very difficult to achieve.

Nowadays, most ecotoxicity preservatives, based on the acute toxicity tests performed on aquatic organisms, include organochloride compounds, isothiazolinones, and quaternary ammonium compounds (QACs). This section characterizes the most controversial examples of preservatives used in cosmetics (Table 1).

### 2.1. Organochloride Compounds

Organochloride preservatives have rather varying success in the marketplace, ranging from highly controversial and almost banned molecules to those that are very popular and successful. Preservatives from this group include chemicals, such as triclocarban or chloroxylenol. Triclocarban [1-(4-Chlorophenyl)-3-(3,4-dichlorophenyl)urea] (TCC) is a trichlorinated, binuclear phenylurea pesticide that has been globally used as an ingredient in disinfectants, deodorants, soaps, toothpastes, and mouthwashes. Its concentration in products at 0.2% has been approved by the European Union (EU) [29]. TCC has been made and marketed on a massive scale since 1957 and its annual consumption reached 227–454 tons in the USA [30,31,32]. Most commercially obtainable TCC is available in a solid form as a white to off-white crystalline powder with a slight aromatic odor. TCC’s mechanism of action is unknown; however, it is thought to be comparable to that of triclosan (TCS, another antiseptic active component commonly found in PCPs), which has a similar structure [33]. TCS exhibits a biostatic and biocidal efficacy against Gram-positive and Gram-negative bacteria and fungi, as well as against viruses. It permeates the bacterial cell wall and targets multiple cytoplasmic and membrane sites, including RNA synthesis and the production of macromolecules [34]. TCS also blocks the synthesis of fatty acids through the inhibition of enoyl reductase but has no effect on bacterial spores [34,35,36].

Chloroxylenol [4-Chloro-3,5-dimethylphenol] (PCMX) is an antibacterial agent that has been applied to disinfectant products in the United States since the 1950s, such as in liquid soaps, hand washing liquid, solutions used in hospitals to clean surgical instruments, etc. [37]. PCMX is a white to off-white crystalline powder soluble in alcohol, ether, benzene, terpenes, fixed oils, and solutions of alkali hydroxides and it is sparingly soluble in water. Some antibacterial ingredients, such as triclosan in PCPs, have been banned in some countries, leading to an increase in the use of antibacterial alternatives, such as PCMX. The use of PCMX as an antibacterial ingredient in PCPs has been on the rise [38]. PCMX is the active ingredient in Dettol disinfectant solution and has unique in vitro and in vivo antimicrobial activity against Gram-positive and Gram-negative bacteria, fungi, algae, and viruses. The main mechanisms of its action are altering the integrity of membrane proteins, changing the permeability of the cell wall, and disrupting its biological processes. The maximum concentration of PCMX in ready-for-use preparation is 0.5% [39]. The worldwide SARS-CoV-2 pandemic has forced a significant increase in the number of used disinfectants. Increased hygiene standards became applicable not only in hospitals but also in households. Singapore’s National Environment Agency (NEA) prepared a list of active substances that are effective against the virus. One of the active components is chloroxylenol, which has a concentration of 0.12% [40].

### 2.2. Isothiazolinones

Isothiazolinones are a group of chemicals with antimicrobial effects, which have been used as preservatives in cosmetics, in other consumer products, and in chemical products for occupational use since the 1970s. These compounds are heterocyclic derivatives of 2H-isothiazolin-3-one chemicals containing vicinal sulfur and nitrogen atoms. Isothiazolinones exhibit excellent broad-spectrum antimicrobial activity against Gram-positive and Gram-negative bacteria and fungi at low concentrations and over a wide range of pH values. Due to the sulfur heterocycle, they react with nucleophilic molecules, bind to the thiol groups of proteins and, consequently, inhibit the activity of enzymes that are essential for growth and metabolism, which leads to microbial cell death after a few hours of contact. A number of isothiazolinones exist, which all may be applied to products for occupational use, while only two have been permitted in cosmetic products. These preservatives are often masked under the chemical names of their mixtures: the mixture of methylchloroisothiazolinone with methylisothiazolinone (MCI/MI) in 3:1, also often named by its tradename Kathon™. The cosmetics industry commonly includes Kathon in a wide range of both rinse-off and leave-on formulations, such as shampoos, gels, and hair and skin care products. Methylisothiazolinone (MI, MIT) is one of the most used preservatives in shampoos and one of the most effective. In March 2013, MIT was called the “Allergen of the Year” and its usage has been self-restricted to rinse-off applications. In consumer products other than cosmetics, different isothiazolinones are used, including benzisothiazolinone (BIT) and octylisothiazolinone (OIT) [27,41,42,43,44,45,46].

### 2.3. Quaternary Ammonium Compounds

Quaternary ammonium compounds (QACs) mainly represent cationic surfactants. In terms of chemical structure, quaternary ammonium compounds belong to ionic compounds that contain four organic groups in the molecule and are associated with nitrogen atoms (including three covalent and one coordination bonds). The antimicrobial activity of QACs depends on the length of the N-alkyl chain, which confers lipophilicity. Benzalkonium chloride (BAC) is one of the most important quaternary ammonium compounds and has been used since 1935 as an antimicrobial additive in various cosmetic preparations at a concentration of 0.1% as well as in pharmaceutical preparations. BAC is a mixture of alkylbenzyl dimethylammonium chlorides with several analogues varying in the length of the aliphatic alkyl chain. In commercial preparations, the aliphatic alkyl chains possess lengths of 12, 14, and 16 carbon atoms. The optimum activity against Gram-positive bacteria and yeast is obtained with chain lengths of 12 to 14 alkyls, while the optimum activity against Gram-negative bacteria is obtained with chain lengths of 14–16 alkyls. Compounds with N-alkyl chain lengths <4 or >18 are virtually inactive. The sensitivity of micro-organisms to the action of QACs also depends on the concentration. At low concentrations (0.5–5 mg L^−1^), these compounds biostatically act on most bacteria, mycobacteria, spores, fungi, and algae. At medium concentrations (10–50 mg L^−1^), they show a biocidal effect on bacteria and fungi while, even in very high concentrations, they do not have a biocidal effect on spores, mycobacteria, and prions. Quaternary ammonium salts also act on lipid-enveloped viruses, including HIV (human immunodeficiency virus) and HBV (hepatitis B virus). Thanks to their high antiviral activity, products containing QAC as the active ingredient have been included in the List N: Disinfectants for Use Against SARS-CoV-2, where there are over 500 products meeting the US EPA criteria for the control of SARS-CoV-2 [47,48]. QACs are frequently used at high levels in hair washing and conditioning products because of their anti-static and softening properties. These compounds are widely used not only in cosmetics but also in agriculture (fungicides, pesticides, and insecticides), in health care (medicines), and in industry (anti-corrosive and anti-electrostatic agents) [25,47,49,50,51,52].

## 3. Occurrence and Ecotoxicity

### 3.1. Occurrence in Sewage and Sludge

**Figure 1:**
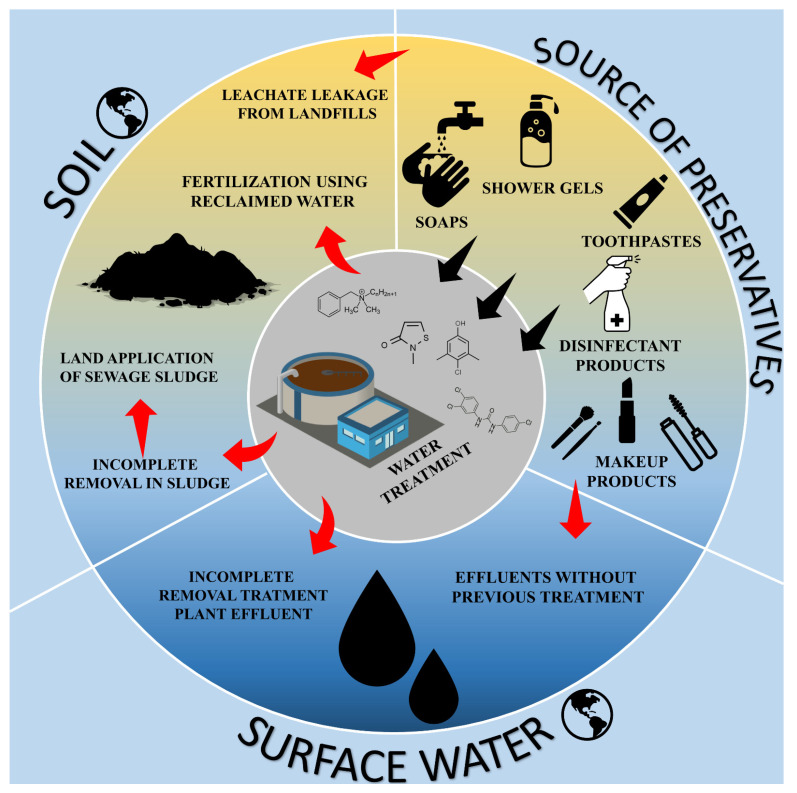
Environmental pathways for cosmetic preservatives.

The extensive application of PCPs in industrial and consumer products has led to the widespread contamination of the environment. There are several direct and indirect pathways through which preservatives can be introduced into the aqueous environment (Figure 1). Municipal wastewater with residues of xenobiotics is identified as the major route responsible for water contamination with micropollutants, such as preservatives and different PCPs. All four preservatives were reported in WWTPs’ influents, effluents, and sludge samples between 2011 and 2021. According to the conducted research, the described preservatives were found in different geographical regions of the world, mainly in some Asian, North American, and European countries. The concentrations of four preservatives significantly ranged from below the limit of quantification (LOQ) to a few tens of micrograms per liter or kilogram dry weight and usually showed high detection rates (Table 2). The analytical results from WWTPs in the world, revealed that the highest influent concentration level was obtained for chloroxylenol with the detection frequency of 80% and a maximum of 404.09 µg L^−1^ in the sewage treatment plants (STPs) in the Tianjin region in China. In this case, PCMX was identified only in influents, which indicates the high efficiency of the traditional activated sludge and anaerobic-anoxic-oxic (A^2^O) techniques, which are two commonly used treatment technologies in these STPs [53]. The removal rates of PCMX in some European (United Kingdom) WWTPs were approximately 98% and the effluent concentrations of PCMX were lower than 140 ng L^−1^ [54]. There is a lack of information concerning chloroxylenol concentrations in WWTP sludge.

BAC was found in lower influent concentration levels in comparison to PCMX, with the highest concentration of 43.5 µg L^−1^ detected for BAC-C12 in Korea [55]. Additionally, high levels were detected in European countries, such as Sweden, the Netherlands, and France, where BAC-C12 ranged from 200 to 29,655 ng L^−1^ with a detection frequency of 100% [56,57,58]. Among the BAC homologs, BAC-C14 was also highly abundant, with a maximum concentration of 8903 ng L^−1^ in WWTPs in Sweden [56]. BAC was not only found in the liquid phase but it was also detected in the suspended particulate phase, where the concentrations were higher than in the liquid phase due to their high adsorption on suspended particulate matter. The sludges in WWTPs in Sweden were mainly contaminated by BAC-C12 and BAC-C14 with a detection frequency of 100% and maximum concentrations of 89,000 and 60,000 ng g^−1^ dw, respectively [56,59]. In effluents, the levels of BACs were much lower than in influents, ranging from below detection limit to 500 ng L^−1^ with a removal rate of 98% in China WWTPs [56,59,60].

Among the discussed preservatives, the most research was focused on TCC. For example, the highest concentrations of 515–10,000 ng L^−1^ with a detection frequency of 100% were reported for TCC in influents from India [61,62,63]. In China, Chen et al. reported a mean concentration of 267 ng L^−1^ of TTC while, in another study from China, Sun et al. reported influents in concentrations ranging from 4.7–76.2 ng L^−1^ [64,65]. High concentrations in influents were also observed in North America, where the mean concentrations reported by Hedgespeth et al. were 4566 and 4644 ng L^−1^ with a detection frequency of 100% [66]. Similarly, Lozano et al. reported a mean concentration of 4920 ng L^−1^ in the USA [67]. On the other hand, in the study by Oliveira et al., the mean concentrations of TCC in different WWTPs in the USA ranged from 210 to 390 ng L^−1^ [68]. In European countries, influent concentrations of TCC reached a maximum of 140 ng L^−1^ [69]. TCC elimination effectiveness varied between 11.4 and 97% in WWTPs. However, due to its high octanol-water distribution coefficients, TCC was more often detected at a high concentration in the primary sludge from WWTPs and the high removal rates are accounted for by its attachment to solids [67,70,71]. The sludge was highly contaminated by TCC in India WWTPs. Subedi et al. reported high concentrations of TCC in sewage sludge samples collected in 2012, where preservative concentrations ranged from 5570 to 6740 ng g^−1^ [62]. In the next study by Subedi et al., in samples collected in 2013, the concentration of TCC was significantly higher with a maximum of 10,000–28,000 ng L^−1^ and a detection frequency of 100% [63]. High concentrations of TCC in sewage sludge were reported in Canada and China, in the range of 1200–8900 ng g^−1^ dw and 887–8450 ng g^−1^, respectively [64,72,73,74]. The effluent concentrations of triclocarban were generally lower than µg L^−1^, except for a sample from India WWTP, where the mean concentration reached 5860 ng L^−1^ [62]. In most WWTPs in the world, the detection frequency of TCC in effluents is approximately 100%.

There is a lack of information concerning MIT concentrations in WWTPs in the world. Limited data showed the presence of MIT in plant wastewater in Poland at a concentration of 1210 ng L^−1^ [20]. Paijens et al. detected MIT in French WWTP influent and effluent samples in the range of 350–860 and 39–110 ng L^−1^, respectively [59]. In another sampling campaign in French WWTPs, Paijens et al. revealed MIT at median concentrations of 620 ng L^−1^ and 150 ng L^−1^ in influents and effluents, respectively [58]. The removal of this preservative ranged from 55 to 89%. There are no data on the occurrence of MIT in other countries and sewage sludge samples.

**Table 2:** Occurrence of preservatives in the wastewater treatment plants.

Compound	Region	Location	Date/n ^a^	Influent	Effluent	Sludge	Analytical Method	Reference
TCC	Asia	China	2008/5		390 ng L^−1 b^		LC-MS/MS	[75]
					183 ng L^−1^			
		China	Unknown/3	267 ng L^−1^	36.6 ng L^−1^	887 ng g^−1^	UHPLC-MS/MS	[64]
		South Korea	2011/40			<LOQ ^c^–1260 ng g^−1 d^ (100%) ^e^	HPLC-MS/MS	[61]
						1630–5090 ng g^−1^ (100%)		
						362–6930 ng g^−1^ (100%)		
		India	2012/unknown	515 ng L^−1^	22.4 ng L^−1^	5620 ng g^−1^	HPLC-ESI-MS/MS	[62]
				933 ng L^−1^	457 ng L^−1^	6740 ng g^−1^		
				8880 ng L^−1^	5860 ng L^−1^	8460 ng g^−1^		
				1150 ng L^−1^	48.4 ng L^−1^	5570 ng g^−1^		
				2100 ng L^−1^	375 ng L^−1^			
		China	2014/12	4.7–76.2 ng L^−1^ (100%)	27.6–109 ng L^−1^ (100%)	1130–2180 ng L^−1^ (100%)	LC-QqQ MS	[73]
		Singapore	2015/4	423.9–933.9 ng L^−1^ (100%)	143.1–214.5 ng L^−1^ (100%)		UHPLC-MS/MS	[71]
					49.1–263.9 ng L^−1^ (100%)			
		India	2013/7	1300–4300 ng L^−1^ (100%)	300–860 ng L^−1^ (100%)	13,000–28,000 ng L^−1^ (100%)	HPLC-MS/MS	[63]
				1200–10,000 ng L^−1^ (100%)	215–358 ng L^−1^ (100%)	10,000–23,000 ng L^−1^ (100%)		
		China	2009–2014/100			8450 ng g^−1 c^	LC-MS/MS	[74]
	North America	USA	2009–2010/unknown	4566 ng L^−1^ (100%)	617 ng L^−1^ (100%)		HPLC-MS/MS	[66]
				4644 ng L^−1^ (100%)	311 ng L^−1^ (100%)			
		USA	2009/5	4920 ng L^−1^	20 ng L^−1^		LC-MS	[67]
		Canada	Unknown/36	14–270 ng L^−1^ (86%)	3.1–33 ng L^−1^ (92%)	1200–8900 ng g^−1^ dw ^h^	LC-MS/MS	[72]
		USA	2013/6		0.19 µg L^−1^		LC-MS/MS	[68]
			2013/5	0.21 µg L^−1^	0.05 µg L^−1^			
			2013/8	0.37 µg L^−1^				
			2013/8	0.39 µg L^−1^	0.07 µg L^−1^			
			2013/8	0.21 µg L^−1^				
	Europe	France	2010/2	97–140 ng L^−1^			UPLC-MS/MS	[69]
		Ireland	2015/16			0.08 µg g^−1^	LC-MS/MS	[32]
PCMX	Asia	China	unknown	404.09 µg L^−1^ (80%)	n.d. ^f^		GC-MS	[53]
				16.22 µg L^−1^	n.d.			
				3.68 µg L^−1^	n.d.			
				7.29 µg L^−1^				
	Europe	United Kingdom	Unknown/9		19–140 ng L^−1^		GC-MS	[54]
MIT	Europe	Poland	2018/3	1.21 µg L^−1^			LC-MS/MS	[20]
		France	unknown	860 ng L^−1^	110 ng L^−1^		HPLC-MS/MS	[59]
				430 ng L^−1^	65 ng L^−1^			
				39 ng L^−1^	<LOQ			
		France	2018–2019/6	35–860 ng L^−1^ (100%)	39–350 ng L^−1^ (100%)		HPLC-MS/MS	[58]
BAC	Asia	Korea	2016/unknown				HPLC-MS/MS	[55]
		BAC12		18 µg L^−1^	0.479 µg L^−1^			
				43.5 µg L^−1^	0.014 µg L^−1^			
		China	2016/unknown				UPLC-MS/MS	[60]
		BAC12	Autumn	0.308 µg L^−1^	<MDL ^g^			
			Winter	0.480 µg L^−1^	<MDL			
			Autumn	0.622 µg L^−1^	<MDL			
			Winter	0.650 µg L^−1^	0.010 µg L^−1^			
		BAC14	Autumn	0.121 µg L^−1^	<MDL			
			Winter	0.161 µg L^−1^	<MDL			
			Autumn	0.141 µg L^−1^	<MDL			
			Winter	0.220 µg L^−1^	<MDL			
		China	unknown				HPLC-MS/MS	[76]
		BAC12		1800 ng L^−1^	3.7 ng L^−1^			
				1400 ng L^−1^	6.8 ng L^−1^			
				1300 ng L^−1^	4.8 ng L^−1^			
		BAC14		670 ng L^−1^	1.9 ng L^−1^			
				610 ng L^−1^	3.3 ng L^−1^			
				480 ng L^−1^	2.3 ng L^−1^			
	North America	USA	2018/13				LC-HRMS/MS	[77]
		BAC12			23 ng L^−1^			
		BAC14			216 ng L^−1^			
	Europe	Sweden	unknown				LC-MS/MS	[56]
		BAC10		2–64 ng L^−1^ (100%)	<LOQ-3 ng L^−1^ (12%)	24–210 ng g^−1^ dw (100%)		
		BAC12		1725–29,655 ng L^−1^ (100%)	<LOQ-310 ng L^−1^ (67%)	8800–89,000 ng g^−1^ dw (100%)		
		BAC14		454–8903 ng L^−1^ (100%)	<LOQ-84 ng L^−1^ (58%)	3200–60,000 ng g^−1^ dw (100%)		
		BAC16		<LOQ-1485 ng L^−1^ (88%)	<LOQ-13 ng L^−1^ (6%)	990–4900 ng g^−1^ dw (100%)		
		Netherlands	2014/15	15.5 µg L^−1^	<LOQ		LC-MS/MS	[57]
				10 µg L^−1^	0.5 µg L^−1^			
		France	unknown				UPLC-MS/MS	[58]
		BAC12		200 ng L^−1^	100 ng L^−1^			
				500 ng L^−1^	400 ng L^−1^			
				2000 ng L^−1^	300 ng L^−1^			
		BAC14		<LOQ	<LOQ			
				400 ng L^−1^	200 ng L^−1^			
				300 ng L^−1^	80 ng L^−1^			
		BAC16		200 ng L^−1^	<LOQ			

^a^ Date/n: sampling date and number. ^b^ Mean or median value. ^c^ Limit of quantification. ^d^ Concentration range. ^e^ Detection frequency. ^f^ Not detected. ^g^ Method detection limits. ^h^ Dry weight.

### 3.2. Occurrence in Surface Waters

The deficient removal of preservatives from WWTPs and the following discharge of effluents lead to the contamination of the receiving environments. Concentrations of selected preservatives in surface waters range from ng L^−1^ to tens of µg L^−1^, thus demonstrating the ubiquity of these pollutants. BAC shows much higher concentrations and detection frequencies than other analyzed preservatives. Analyses conducted in water samples, obtained from eight different located ponds in Changsha city in Hunan Province in China, showed a maximum concentration of tetradecyl benzyl ammonium chloride (BAC-C14) of up to 8.1 mg L^−1^ [78]. Li et al. examined the occurrence of cationic surfactants and other pollutants from Songhua River, Second Songhua River, and Nen River, where BAC-C12 was detected in 87% of a total 196 samples, with a maximum concentration of 41 ng L^−1^ and an average concentration of 3.5 ± 5.3 ng L^−1^ [79]. In addition, BAC-C14 was detected in samples with a detection frequency of 98% and with a maximum concentration of 13 ng L^−1^. The authors suggested that the low concentrations of cationic surfactants in surface waters are caused by the limited usage of these chemicals in Northern China. Meanwhile, in Korea, the maximum concentrations of BAC-C12 and BAC-C14 in surface waters reached 35.8 µg L^−1^ and 21.6 µg L^−1^, respectively. In the case of European countries, the analyses of samples from five different sites in the city of Gdańsk (Poland) showed a maximum concentration of hexadecyl benzyl dimethyl ammonium chloride (BAC-C16) of up to 243 µg L^−1^. Additionally, high contents of BAC-C12 and BAC-C14 were observed, with maximum concentrations of 99.6 µg L^−1^ and 157 µg L^−1^, respectively [80]. Ruman et al. examined the occurrence of five cationic surfactants from Kłodnica River in Poland in four seasons [81]. BAC-C12, BAC-C14, and BAC-C16 were detected in water samples, with maximum concentrations of 99.1 µg L^−1^, 76.1 µg L^−1^, and 89.4 µg L^−1^, respectively. All of the maximum concentrations were detected in the cold season.

Studies on the occurrence of chloroxylenol in receiving aquatic environments in the last ten years were conducted only in Asian countries. Recent research on the occurrence of PCMX in surface water in China was conducted by Tan et al. [38]. Nine surface water samples were collected from two urban streams, Pearl River located in Guangzhou, and an outlet of the STP. The PCMX concentrations in the urban streams reached 8.89 µg L^−1^; in Pearl River, they ranged from 1.62 to 3.60 µg L^−1^; and in the outlet of the sewage treatment plant, the average was 2.45 µg L^−1^. Dsikowitzky et al. estimated the concentrations of lipophilic organic contaminants of surface water in Jakarta (Indonesia) in seven locations, in two of which PCMX was detected in the range of 20–30 ng L^−1^ [82]. A year before, the same team examined 18 spots in the rivers of Jakarta city that go into Jakarta Bay. PCMX occurred in 13 spots in concentrations ranging from 60 to 1200 ng L^−1^ [83]. The occurrence of PCMX in another Asian country was detected by Kimura et al. in Tokushima city [84]. Out of four river samples, PCMX was detected only in one from the autumn sampling campaign at a concentration of 17.8 ng L^−1^.

Triclocarban is one of the most studied preservatives in the world due to its environmental impact. Many studies describe its occurrence in receiving environments. One of them was conducted by Vimalkumar et al., where concentrations of triclocarban in three major rivers in India were analyzed [29]. Sampling was conducted during wet and dry seasons from 29 locations. The highest concentration of TCC was detected in Karei River ranging from 8 to 1119 ng L^−1^. In Thamiraparani River and Vellar River, the average concentrations were 55.6 and 25.3 ng L^−1^, respectively. TCC was detected in all tested samples. TCC was also found in another Indian river (Torsa River) at a maximum concentration of 77 ng L^−1^ [85]. On the other hand, TCC was not detected in another Indian river: Arkavathi River [86]. Slightly lower concentrations of TCC were noticed in samples collected from Sri Lanka aquatic environments. Triclocarban was detected in 100% of them, with a maximum concentration of 31 ng L^−1^ [87]. High concentrations of preservatives in the surface water were detected in samples closer to the outfall of effluents from WWTPs, pointing to the major source of these pollutants. Juksu et al. examined the total emission of TCC from WWTPs based on the estimated consumption in Thailand [88]. TCC was one of the biggest pollutants, with >30 tons/year for the entire country. Moreover, TCC had the highest concentration in receiving riverine environments (4030 ng L^−1^) and in sea water in the coastal environments of Pattaya city (248 ng L^−1^). The occurrence of TCC in China river environments was examined in Songhua River, Second Songhua River, and Nen River [79]. The total concentrations of TCC in river water ranged from below detection limit to 27 ng L^−1^ with a detection frequency of 96%. Comparatively, the levels of TCC in sea water were in the range of <LOD-13.2 ng L^−1^ in the coastal areas of Zhejiang in the East China Sea and Xiamen Bay [89,90]. TCC was also detected in North American surface waters in concentrations ranging from 2.5–102 ng L^−1^ [91,92]. TCS was reported to occur in low concentrations in European countries, e.g., in the Italian, Spanish, Romanian, and Polish surface waters (>0.8 ng L^−1^; 0–15 ng L^−1^; 3 ng L^−1^; 0.6–54 ng L^−1^; and 5 µg L^−1^) [93,94,95,96,97]. TCC was also found in the samples from the western basins of the Mediterranean Sea and the North Sea, in concentrations ranging from 0.0036 to 0,07 ng L^−1^ [98,99].

There are few literature data concerning the occurrence of MIT in surface water. Paijens et al. described contamination of Paris wastewater by MIT at concentration 14 ng L^−1^ [59]. MIT was also a subject of research by Nowak et al., who examined the contamination of Vistula River (Poland), where the preservative was not detected [20].

### 3.3. Occurrence in Surface Water Sediments

There are only a few papers on the presence of preservatives in sediments. Accessible research merely concerns two compounds discussed in this work: triclocarban and benzalkonium chloride. Due to high adsorption and resistance to microbial removal, BAC may be detected in surface water sediments, though at significantly lower levels than in sewage sludge samples [100]. The most contaminated samples were from Hudson River Estuary, New York, USA, with maximum concentrations of 8900, 4000, 3800, and 1000 ng g^−1^ detected for BAC-C14, C18, C16, and C12, respectively [101]. Comparing the median concentrations of different BAC homologs, the highest median value was determined for BAC-C18. The authors suggested that BAC-C18 is most used in PCPs that may reach WWTPs. Relatively lower concentrations of BAC homologs were determined in the rivers of China (Songhua River, Second Songhua River, Nen River, Pearl River, and Zhujiang River). Concentrations for total BAC in the Chinese rivers were measured by Li et al. and ranged from 49.3 to 1530 ng g^−1^ [102]. The average concentrations of BAC-C12 and BAC-C14 were determined by Li et al. and were 1 ± 1.6 ng g^−1^ dw and 0.44 ± 0.69 ng g^−1^ dw, respectively [79]. Domestic wastewater is the main source of biocides in rivers and its concentrations depend on the economic level of the area, the population of cities along the river, the everyday activities of the populace, the different physical and chemical properties of various sites, and the type of surface water system receiving treated wastewater. However, the area of influence of WWTPs and the location of the sampling site seems to dictate the contamination levels. The levels of TCC in surface water sediments in the USA were measured by Venkatesan et al. and Maruya et al. [103,104]. Samples collected from Minnesota freshwater sediments were contaminated by TCC from 5 to 822 ng g^−1^ dw. TCC concentrations in sediment collected from near the area of influence of WWTPs’ discharge were higher than those observed in any river or creek sediments downstream of WWTPs discharges [103]. In Southern California, the highest detectable concentration of TCC in river sediments was 183 ng g^−1^ dw. It was higher than in estuarine sediments, which again may indicate the influence of physicochemical properties, i.e., pH or salinity, on the fate of biocides in the natural environment [104]. Other researchers reported the occurrence of TCC in sediments from European surface water sediments. The concentrations ranged from 0.7 to 6.91 ng g^−1^ in the upstream and downstream of River Lambro in Northern Italy, and in Lake Lugano and Greifensee in Switzerland, they reached 7 ng g^−1^ dw [105,106]. In another study, an analysis of 19 grab samples of sediments from the Albufera Natural Park in Spain showed the occurrence of TCC at a concentration below 10 ng g-1 with a detection frequency of 42% [97]. Elevated levels of TCC were also found in sediments from Asian surface waters. The mean concentration of TCC detected in Thao Praya River in Bangkok, Thailand was 3370 ng g^−1^ [88]. A widespread occurrence of TCC was reported in Pearl River Delta, at concentrations ranging from 0.53–103 ng g^−1^. The concentrations of TCC were lower in the adjacent tributaries than in the mainstream, suggesting that municipal sewage is the main source of contaminants [107]. The lowest concentrations were observed in coastal areas of Zhejiang in the East China Sea. Detection frequency was 100% and concentrations ranged from 0.12 to 6.6 ng g^−1^ [90].

### 3.4. Occurrence in Soil

The available literature data describing the concentrations of preservatives in soil mainly concern agricultural soils. The widespread use of sewage sludge, solid waste, or reclaimed water for soil fertilization and irrigation poses a serious risk of soil contamination with residual chemicals that are not completely removed during the wastewater treatment process. In addition, the use of pesticides, which may contain preservatives, is also a source of soil contamination. Despite the concerns, few data are available. As with surface water sediments, the majority of papers describe BAC and TCC concentrations. Both described compounds due to the high log organic carbon-water partitioning coefficient (K_oc_), indicating strong trends to accumulate in the soil [75,108].

A study revealed that the BAC homologs are able to persist in soils for half a year after application. With the use of liquid chromatography-tandem mass spectrometry (LC-MS/MS), for the first time, Kang and Shin showed BAC concentrations in soil [109]. The total BAC was detected in Korean soil samples from sentry posts, cattle farms, and migratory bird habitats in concentrations ranging from 0.001 to 28.5 mg kg^−1^. BAC-C12 occurred in all tested soil samples. Scarce water resources in arid and semi-arid regions of the Earth caused reclaimed wastewater to be used for the irrigation of soil. Chen et al. reported a range of concentrations from 8.5 ± 11.9 to 105 ± 38.9 µg kg^−1^ dw for TCC in Hebei (China) at different soil depths irrigated with reclaimed wastewater and evaluated the TCC half-life, which was 108 days [53]. TCC was also detected in biosolid-amended soils in Chinese provinces (Zhejiang, Hunan, and Shandong) at concentrations ranging from 111 to 1584 µg kg^−1^ dw. The authors observed that the concentrations of TCC in soils fertilized several times with biosolids were significantly higher than those of a single application. Moreover, the occurrence of TCC in soil samples after three years since the first application of biosolids implies a tendency of TCC to persist in the soil. TCC concentrations in the control plots, free from biosolid applications, were below the detection limit or very low, suggesting that the occurrence of contaminants, such as TCC, is caused by the use of biosolids [110]. In another study, TCC was also detected in biosolid-amended soil in North America in the state of Idaho. Tested soil samples were treated with biosolids for seven years; however, the TCC concentration range was lower than in China (14.8–27.3 ng g^−1^ dw). Similar results were received by Viglino et al. and Negahban-Azar et al. in Canada (13 ± 2–53 ± 9 ng g^−1^) and the USA (2.5–9.1 µg kg^−1^) [111,112], respectively. The lower levels of contaminations in these studies could result from different physio-chemical properties of soil, an alternative method of wastewater treatment, and different amounts of biosolid application [113]. The data from another state (Virginia), collected from commercial farms by Lozano et al., showed that the highest measured concentration of TCC (131.9 ± 76.1 ng g^−1^ dw) was observed in fields that received biosolids multiple times [114]. A lower concentration (107.1 ± 43.7 ng g^−1^ dw) was observed in soil from the field, which received a single application of biosolids. Additionally, a trace quantity of TCC (<19.7 ± 3.7 ng g^−1^ dw) was observed in soil never fertilized with biosolids. Moreover, the authors evaluated TCC concentrations seven and eight years after biosolid application, which were at 45.8 ± 6.09 and 72.4 ± 15.3 ng g^−1^ dw, respectively. The last analysis proves that TCC has high persistence in soil.

Soil can be contaminated not only through fertilization or irrigation but also through general human activity. One of the reasons for soil contamination with preservatives may be inadequate solid waste disposal. The research carried out by Nowak et al. showed the presence of MIT in sand samples collected in the summer season from the Baltic Sea coast [20]. In the publication, the authors suggested that sand contamination may be due to the excessive use of skin-protecting agents against UV radiation. MIT concentrations in sand samples ranged from 2.19 ± 0.47 to 4.48 ± 1.04 µg kg^−1^.

There are a lack of literature data describing the occurrence of chloroxylenol in soil samples.

### 3.5. Aquatic Toxicity

**Figure 2:**
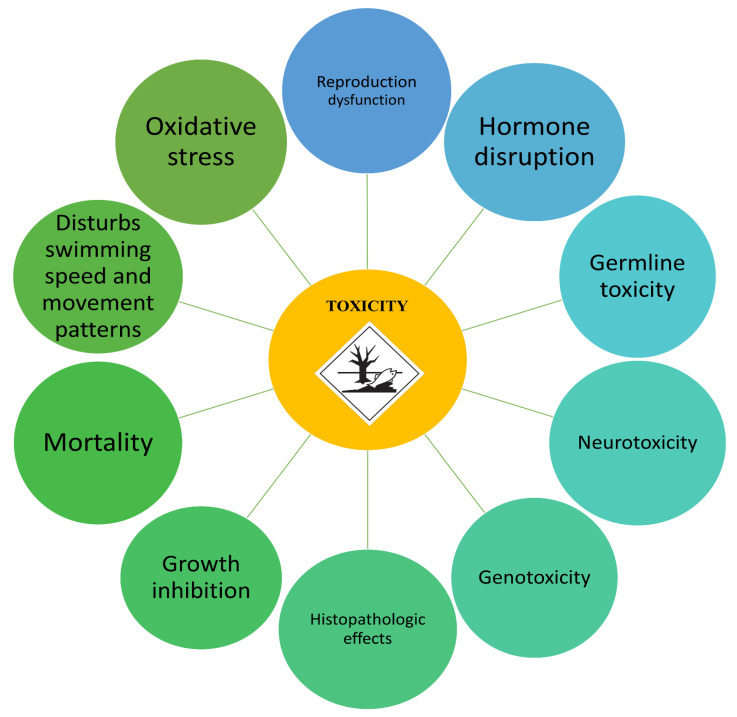
Toxicity of cosmetic preservatives.

The toxic effects of preservatives on non-target organisms were studied on several model species from different trophic levels. Some of the acute and chronic toxicity effects for triclocarban, chloroxylenol, benzalkonium chloride, and methylisothiazolinone are listed in Table 3. Histopathological alterations, modification of proteins, neurotoxicity and genotoxicity, reproduction and structural abnormalities, embryotoxicity, and endocrine disturbance have been observed as a result of preservative toxicity in aquatic organisms (Figure 2).

#### 3.5.1. Triclocarban

The acute toxicity of triclocarban for water flea (*Daphnia magna*) immobility was described by an EC_50_ (effective concentration, 50%) of 5.9 µg L^−1^ at 24 h [115]. Sreevidya et al. investigated the ecotoxicity of triclocarban on two aquatic organisms, nematode *Caenorhabditis elegans* and zebrafish *Danio rerio* [116]. TCC below 1 mg L^−1^ could result in disorders in reproduction and an abridged lifespan of *C. elegans*. Moreover, TCC induced germline toxicity in the exposed worm, manifested by the increased occurrence of the “green eggs” phenotype. The measured median lethal concentration (LC_50_) was 0.91 mg L^−1^ for *C. elegans*. TCC at 0.1 mg L^−1^ and 0.5 mg L^−1^ induced larval mortality of zebrafish after 72 h post-fertilization (hpf). Among the many different toxic effects of TCC, an interesting observation is that the xenobiotics caused developmental neurotoxicity in the *Danio rerio* embryos. Zebrafish embryos exposed to 0.1–0.5 mg L^−1^ showed abnormalities in secondary motor neurons. The TCC developmental toxicity in *D. rerio* was also confirmed in a study by Dong et al. [117]. The calculated LC_50_ value for *D. rerio* was 215.8 µg L^−1^. Furthermore, the obtained results showed that exposure to 133.3 µg L^−1^ TCC influenced thyroid hormone activity and disturbed the expression of genes. The toxicity potential of TCC, at low concentrations, was evaluated for silver catfish *Rhamadia quelen*. Environmental concentrations of TCC induced sublethal effects, such as deformities of embryos, oxidative damage, and neurotoxic effects [118]. Jimoh and Sogbanmu observed the dose-dependent gill histopathological alterations in *Clavias gariepinus* as well as embryotoxic effects [119]. Three typical freshwater algae *Chlorella vulgaris*, *Scenedesmus obliquus*, *Chlorella pyrenoidosa* were less vulnerable to triclocarban than fish and nematodes, with a 96 h EC50 of 8.474, 9.11, 8.76 mg L^−1^, respectively, based on the growth inhibition. Triclocarban significantly altered the content of chlorophyll α and disturbed the activity of peroxidase and superoxide dismutase enzyme, destroying the antioxidant functions of cells. Moreover, the growth in the malondialdehyde content indicated elevated stress levels in algae [120].

#### 3.5.2. Chloroxylenol

Won et al. reported that the LC_50_ value of PCMX for the rotifer *Brachionus koreanus* was 24.264 mg L^−1^ [121]. The population growth and reproduction ability of rotifers were significantly inhibited in response to PCMX in a dose-dependent manner. Swimming speed as well as movement tracking of the tested organisms were disturbed after PCMX exposure. The occurrence of this disinfectant in the *B. koreanus* environment caused an increase in ROS (reactive oxygen species) generation. PCMX showed mutagenic activity in the DNA of (Rainbow trout) erythrocytes [122]. Similar to TCC, PCMX disturbed the reproduction and lifespan of *C. elegans* and showed germline toxicity and neurotoxicity toward *D. rerio*. Moreover, PCMX in *D. rerio* induced embryonic malformations, for example, body curvature, and caused an increase in mortality. The reported LC_50_ value of PCMX to *C. elegans* after 24 h of exposure was 31.8 mg L^−1^ [116]. The acute values of chloroxylenol towards whirligig beetles (*Orectogyrus alluaudi*) for mortality were reported at LC_50_ values of 21.587, 16.744, 11.638, and 7.819 mg L^−1^ at 24, 48, 72, and 96 h, respectively. The observed increase in mortality of *O. alluaudi* was dependent on the concentration and exposure duration. These results suggest that *O. alluaudi* has a higher vulnerability toward PCMX than other invertebrates [123]. However, the 48 h LC_50_ for *Daphnia magna* was lower and reached 8.78 mg/L [124]. These differences in the sensitivity of aquatic organisms (Table 3) can be associated with differences in biochemical responses, exposure routes, and psychological responses.

#### 3.5.3. Methylisothiazolinone

Different species of invertebrates have different sensitivities towards methylisothiazolinone (Table 3). The short-term median lethal concentration values (LC_50_) of MIT on three freshwater invertebrates, *Daphnia similis*, *Dugesia japonica*, and *Neocaridina denticulata,* varied from 1.83 to 198.34 mg L^−1^ at 24 h [125]. In contrast, EC_50_ values for MIT for *D. magna* were reported at a concentration of 510 µg L^−1^ [126]. Moreover, MIT at a level of 15 µM caused alterations in regeneration and wound healing in planaria (*D. japonica*) as well as defects in neuromuscular and epithelial integrity [127]. Wang et al. evaluated the tolerance of microalgae (*Scenedesmus sp*. LX1) to MIT by testing their growth inhibition [128]. The results indicated that MIT caused the growth inhibition of microalgae by photosynthesis disturbance. The EC_50_ value for these aquatic organisms was 1 mg L^−1^. Capkin et al. confirmed the genotoxic and histopathologic effects of MIT on rainbow trout, causing DNA damage in red blood cells and up-regulation of all studied genes [125]. In addition, MIT at a concentration of 300 µg L^−1^ disturbed the ability to hatch and the survival of zebrafish larvae and caused the deregulation of thyroid hormone gene expression, resulting in a reduction in the content of triiodothyronine and thyroxine in the whole body of *D. rerio* larvae [129].

#### 3.5.4. Benzalkonium Chloride

Studies regarding the toxicity of benzalkonium chloride have been focused on fish, algae, and invertebrates, such as daphnids and rotifers. The 48 h acute toxicity of BAC to *D. magna* (EC_50_) is 41.1 µg L^−1^, which is significantly lower than its toxicity toward microalgae *Phaeodactylum tricornutum* (EC_50_: 131.9 µg L^−1^), *Tisochysis lutea* (EC_50_: 86 µg L^−1^), and *Pseudokirchneviella subcapitata* (EC_50_: 255 µg L^−1^) [55,130,131]. The hazardous potential of BAC toward aquatic environments was evaluated by Qian et al., who analyzed the toxicity of BAC on freshwater cyanobacteria *Microcystis aeruginosa* [132]. All of the examined BAC-C12 concentrations strongly inhibited cyanobacteria growth, with the 96 h EC_50_ value identified as 3.61 mg L^−1^. Moreover, the exposition of *M. aeruginosa* to BAC-C12 resulted in the inhibition of photosynthetic efficiency by disturbing the chlorophyll-protein-lipid structure and the photosynthetic organelle. The toxicity effects of BAC were also manifested via an increase in oxidative stress and greater permeability of cell membranes. Therefore, it can be suggested that BAC-C12 might enhance the release of microcystins by *M. aeruginosa* and increase its level in the aquatic environment, causing a higher risk for aquatic ecosystems. Similar to chloroxylenol, BAC inflects antioxidant enzymatic activities and the level of ROS and disturbs swimming speed and movement patterns in *B. koreanus* [121]. Some in vitro and in vivo studies have examined the endocrine-disrupting effects of benzalkonium chloride. At concentration of 3 µg L^−1^, BAC was reported to possess endocrine-disrupting properties by Kim et al. in an in vivo assay using the measurement of vitellogenin gene transcription, which is a biomarker of estrogenic activity in male *Oryzias latipes* fish [55]. In another study, the molecular response to long-term exposure to BAC was analyzed with the use of a proteomic approach. BAC showed interactions with proteins responsible for the endocrine and nervous systems, oxidative stress, signaling pathways, cellular proteolysis, and cytoskeleton in *O. latipes* [133].

**Table 3:** Aquatic toxicity values for preservatives.

Compound	Species	Effect	Duration	Endpoint	Value	References
TCC	*Daphnia magna*	Immobility	48 h	EC_50_	5.9 µg L^−1^	[115]
	*Daphnia magna*	Mortality	96 h	LC_50_	0.087 µM	[134]
	*Daphnia similis*	Immobility	48 h	EC_50_	0.044 µM	[135]
	*Pseudokirchneriella subcapitata*	Growth inhibition	72 h	IC_50_^a^	1.01 µM	[135]
	*Chlorella vulgaris*	Growth inhibition	96 h	EC_50_	8.474 mg L^−1^	[120]
	*Scenedesmus obliquus*	Growth inhibition	96 h	EC_50_	9.11 mg L^−1^	[120]
	*Chlorella pyrenoidosa*	Growth inhibition	96 h	EC_50_	8.76 mg L^−1^	[120]
	*Clarias gariepinus*	Fingerlings mortality	96 h	LC_50_	41.57 mg L^−1^	[119]
	*Clarias gariepinus*	Embryos mortality	24 h	LC_50_	46.08 mg L^−1^	[119]
	*Clarias gariepinus*	Hatching	26 h	EC_50_	41.93 mg L^−1^	[119]
	*Caenorhabditis elegans*	Reproduction	96 h	EC_50_	0.38 µmol L^−1^	[136]
	*Caenorhabditis elegans*	Growth	96 h	EC_50_	0.66 µmol L^−1^	[136]
	*Caenorhabditis elegans*	Mortality	24 h	LC_50_	0.91 mg L^−1^	[116]
	*Caenorhabditis elegans*	Reproduction	4–6 days	LOEC^b^	0.01 mg L^−1^	[116]
	*Caenorhabditis elegans*	Lifespan	4–6 days	LOEC	0.05 mg L^−1^	[116]
	*Caenorhabditis elegans*	Germline toxicity	24 h	LOEC	0.01 mg L^−1^	[116]
PCMX	*Brachionus koreanus*	Mortality	24 h	LC_50_	24.264 mg L^−1^	[121]
	*Brachionus koreanus*	Mortality	24 h	NOEC^c^	15 mg L^−1^	[121]
	*Daphnia magna*	Mortality	48 h	LC_50_	8.78 mg L^−1^	[124]
	*Caenorhabditis elegans*	Mortality	24 h	LC_50_	31.8 mg L^−1^	[116]
	*Caenorhabditis elegans*	Reproduction	4–6 days	LOEC^b^	1 mg L^−1^	[116]
	*Caenorhabditis elegans*	Lifespan	4–6 days	LOEC	10 mg L^−1^	[116]
	*Caenorhabditis elegans*	Germline toxicity	24 h	LOEC	5 mg L^−1^	[116]
	*Orectogyrus alluaudi*	Mortality	24 h	LC_50_	21.587 mg L^−1^	[123]
	*Orectogyrus alluaudi*	Mortality	48 h	LC_50_	16.744 mg L^−1^	[123]
	*Orectogyrus alluaudi*	Mortality	72 h	LC_50_	11.638 mg L^−1^	[123]
	*Orectogyrus alluaudi*	Mortality	96 h	LC_50_	7.819 mg L^−1^	[123]
	*Orectogyrus alluaudi*	Mortality	24 h	NOEC	6.754 mg L^−1^	[123]
	*Orectogyrus alluaudi*	Mortality	48 h	NOEC	2.789 mg L^−1^	[123]
	*Orectogyrus alluaudi*	Mortality	72 h	NOEC	1.1535 mg L^−1^	[123]
	*Orectogyrus alluaudi*	Mortality	96 h	NOEC	0.5485 mg L^−1^	[123]
MIT	Daphnid	Mortality	48 h	LC_50_	4.7 mg L^−1^	[137]
	Algae	-	96 h	EC_50_	0.4 mg L^−1^	[137]
	Fish	Mortality	96 h	LC_50_	3.8 mg L^−1^	[137]
	*Daphnia magna*	Immobility	48 h	EC_50_	510 µg L^−1^	[126]
	Cell line RTL-W1 from *Oncorhynchus mykiss*	Vitality	48 h	EC_50_	10400 µg L^−1^	[126]
	*Daphnia similis*	Mortality	24 h	LC_50_	1.83 mg L^−1^	[125]
	*Daphnia similis*	Mortality	48 h	LC_50_	0.81 mg L^−1^	[125]
	*Dugesia japonica*	Mortality	24 h	LC_50_	2.36 mg L^−1^	[125]
	*Dugesia japonica*	Mortality	48 h	LC_50_	2.06 mg L^−1^	[125]
	*Dugesia japonica*	Mortality	72 h	LC_50_	1.58 mg L^−1^	[125]
	*Dugesia japonica*	Mortality	96 h	LC_50_	1.54 mg L^−1^	[125]
	*Neocaridina denticulata*	Mortality	24 h	LC_50_	198.34 mg L^−1^	[125]
	*Neocaridina denticulata*	Mortality	48 h	LC_50_	84.48 mg L^−1^	[125]
	*Neocaridina denticulata*	Mortality	24 h	LC_50_	43.82 mg L^−1^	[125]
	*Neocaridina denticulata*	Mortality	48 h	LC_50_	35.36 mg L^−1^	[125]
	*Scenedesmus sp.* LX1	Growth inhibition	72 h	EC_50_	1 mg L^−1^	[128]
BAC	*Daphnia magna*	Immobility	48 h	EC_50_	41.1 µg L^−1^	[55]
	*Oryzias latipes*	Mortality	96 h	LC_50_	246 µg L^−1^	[55]
	*Oryzias latipes*	Mortality	96 h	LC_50_	2.12 mg L^−1^	[133]
	*Phaeodactylum tricornutum*	Growth inhibition	72 h	EC_10_	69 µg L^−1^	[130]
	*Phaeodactylum tricornutum*	Growth inhibition	72 h	EC_50_	131.9 µg L^−1^	[130]
	*Tisochrysis lutea*	Growth inhibition	72 h	EC_10_	57.1 µg L^−1^	[130]
	*Tisochrysis lutea*	Growth inhibition	72 h	EC_50_	86 µg L^−1^	[130]
	*Pseudokirchneriella subcapitata*	Growth inhibition	72 h	EC_10_	0.092 mg L^−1^	[131]
	*Pseudokirchneriella subcapitata*	Growth inhibition	72 h	EC_50_	0.255 mg L^−1^	[131]
	*Pseudokirchneriella subcapitata*	Growth inhibition	72 h	NOEC	0.023 mg L^−1^	[131]
	*Microcystis aeruginosa*	Growth inhibition	96 h	EC_50_	3.61 mg L^-^	[132]
	*Brachionus koreanus*	Mortality	24 h	LC_50_	0.483 mg L^−1^	[121]
	*Brachionus koreanus*	Mortality	24 h	NOEC^c^	0.3 mg L^−1^	[121]
	Cell line RTgill-W1 from *Oncorhynchus mykiss*	Metabolic activity	24 h	EC_50_	1098 µg L^−1^	[138]
	Cell line RTgill-W1 from *Oncorhynchus mykiss*	Membrane integrity	24 h	EC_50_	1628 µg L^−1^	[138]
	Cell line RTgill-W1 from *Oncorhynchus mykiss*	Rybosomal integrity	24 h	EC_50_	690 µg L^−1^	[138]

### 3.6. Soil Toxicity

The aquatic toxicity of preservatives has been studied extensively; however, their toxicity toward terrestrial organisms is also in need of attention as sewage sludge land application has become one of the major soil fertilization methods. Biosolids contaminated by preservatives, together with many other xenobiotics, may be a potential risk to soil organisms. In the study by Yang et al., BAC up-regulated the N fixation gene (nifH) and nitrification genes (AOA and AOB) in the soil and down-regulated the denitrification gene (narG). Moreover, it reduced the variety of soil microbial communities and caused an increased quantity of *Crenarchaeota* and *Proteobacteria* [139]. TCC was demonstrated to decrease the abundance of soil bacteria and reduce the degradation level of pesticides in soil resulting in their persistence in the environment [140]. Ali et al. also proved the activity inhibition of soil microflora by TCC at a concentration of 450 µg g^−1^ [141]. The bioaccumulation of TCC was reported in earthworm (*Eisenia fetida*) tissues at bioaccumulation factor values ranging from 5.2 to 18 g_soil_ g_tissue_^−1^. The calculated LC_50_ value for *E. fetida* was 40 mg kg^−1^ fine sand [142]. The database on the toxicity of preservatives to soil organisms is still fragmentary.

## 4. Microbial Degradation of Preservatives

Human economic activity and progressive urbanization pose a constant threat of environmental contamination with xenobiotics. The presence of cosmetic preservatives in water and soil samples, confirmed by numerous studies, is the cause of many undesirable processes that contribute to the disturbance of the biological balance, as well as the emergence of unfavorable changes at the ecosystem level. Due to the pollution of the natural environment with xenobiotics and their toxic properties, research on their removal has been developing on a large scale and is subject to great interest. However, few literature data that describe the elimination of these preservatives are available. The main way to remove preservatives from the environment is biodegradation carried out by micro-organisms. Biodegradation is a metabolism-dependent process of decomposition of xenobiotics into simpler compounds, taking place with the participation of extracellular and/or intracellular enzymes. This process often transforms the pollutants into simpler compounds that are typically less toxic than the parent compounds. In some cases, biodegradation leads to the mineralization of organic compounds and their degradation into carbon dioxide, water, and/or other inorganic products.

In the scientific literature, few works describe the potential of bacteria and fungi to effectively eliminate triclocarban, chloroxylenol, methylisothiazolinone, and benzalkonium chloride. It should be noted that the process of elimination of xenobiotics, in some cases, is not synonymous with their degradation and detoxification. Table 4 shows the biodegradation of the discussed biocides with the use of micro-organisms.

### 4.1. Triclocarban

Research studies on the microbial degradation of triclocarban are numerous. The usage of sewage sludge, including of TCC residues in agriculture, poses a serious risk to the environment. The application of micro-organisms over composting biosolids could reduce the environmental risks of the use of sewage sludge as a fertilizer. The biodegradation of TCC via the composting of biosolids under high ventilation resulted in a reduction in xenobiotic concentrations by 83.1% over 16 days [143]. The immobilized microbial cells of *Pseudomonas fluorescens* (MC46) on biochar could be used for the effective purification of the sewage from TCC. The yield of the process carried out by immobilized *P. fluorescens* cells was much higher (79.80%) compared to the elimination of TCC by free *P. fluorescens* cells (42.12%) [144]. Similar results were obtained by Taweetanawanit et al., confirming that TCC was eliminated more efficiently by micro-organisms entrapped in barium alginate [145]. Moreover, researchers observed 3,4-dichloroaniline (34DCA), 4-chloroaniline (4CA), and aniline as by-products emerging via hydrolysis, dehalogenation, hydroxylation, and dechlorination, which were characterized by a lower toxicity than the parent compound. Subsequently, aniline may be transformed through deoxygenation into catechol and it is anticipated that catechol may thereafter undergo ring cleavage [145]. The same bacterial strain was used for the bioaugmentation of TCC-contaminated soil, with an elimination efficiency of 74–76%. *P. fluorescens* was able to remove TCC as a sole carbon source leading to its detoxification [146]. 34DCA, 4CA, and 4-chlorocatechol were reported to be the major metabolites present as a result of the bacterial degradation of TCC in *Sphingomonas sp*. YL-JM2C. The formed metabolites and parent compounds were too toxic for the tested strain and inhibited further biodegradation stopping at the level of 35% [147]. Three strains of *Ochrobacterium sp*. (MC22, TCC-1, and TCC-2) were also capable of triclocarban biotransformation under aerobic and anaerobic conditions [148,149,150,151]. Under aerobic conditions, strain MC22 was able to degrade TCC (initial concentration 9.40 mg L^−1^) as a sole carbon and energy source with an efficiency of 78% within 6 days and to produce two intermediates 34DCA and 4CA, which were detoxified [148]. Similar metabolites were observed in strains TCC-1 and TCC-2; however, these micro-organisms were characterized by a higher tolerance to upper concentrations of TCC [149,150,151]. The anaerobic degradation of TCC by *Ochrobacterium sp*. was conducted with acetate as an electron donor. During transformation, 4CA and DCA were formed in all three strains. Only MC22 produced additional aniline [148,149,150]. Moreover, Yun et al. identified a protein accountable for TCC hydrolysis: amidase TccA [149].

### 4.2. Chloroxylenol

Despite several reports describing the ability of micro-organisms to eliminate chloroxylenol, there is little research devoted to the identification of intermediate products formed during biodegradation and analyzing the mechanisms responsible for the course of these processes. Nowak et al. identified two fungal species capable of degrading chloroxylenol [124]. *Cunninghamella elegans* IM 1785/21GP and *Trametes versicolor* IM 373 degraded PCMX with similar efficiencies through different degradation pathways. *C. elegans* removed 70% of PCMX over 120 h of incubation, at an initial PCMX concentration of 25 mg L^−1^, via the generation of two metabolites by dehalogenation, aromatic ring hydroxylation, and methyl group oxidation of the parent compound. *T. versicolor* demonstrated a 79% removal of PCMX over 120 h of incubation via ring opening during hydroxylation, dehalogenation, and oxidation, leading to the formation of three metabolites. The authors suggested that the two different enzyme systems are involved in the initial step of chloroxylenol degradation: cytochrome P450 mono-oxygenases in *C. elegans* and laccases in *T. versicolor*. Furthermore, the metabolites generated by tested micro-organisms have a lower toxicity than the parent compound. Among the micro-organisms demonstrating the ability to eliminate PCMX, the microscopic fungus *Aspergillus niger* was also described in which over 99% of PCMX loss (initial substrate content – 2 mg L^−1^) was shown after 7 days of incubation [152]. Choi and Oh demonstrated that the removal efficiency of chloroxylenol by activated sludge depended on the initial concentration of xenobiotics [153]. PCMX at a concentration 5 mg L^−1^ was eliminated over two months with a yield below 50%. In this research, the authors analyzed the impact of PCMX on the bacterial community structure and isolated two bacterial strains probably able to degrade PCMX: *Sphingobium* and *Luteolibacter*. Based on the literature data, the authors suggested that the biodegradation of PCMX occurs via dehalogenation and ring hydroxylation.

### 4.3. Methylisothiazolinone

Little is known about the biodegradation of methylisothiazolinone by micro-organisms. Most often, only the initial stages of the biotransformation of this preservative are known. The few micro-organisms that exhibit the ability to metabolize MIT mainly include various species of filamentous fungi [20,154]. The ability of the ligninolytic fungus *Phanerochaete chrysosporium* to biodegrade this biocide over a 48 h incubation in liquid culture under aerobic conditions was described. The tested micro-organism was able to completely eliminate MIT at a concentration of 50 µg L^−1^ and 30 mg L^−1^ within 12 h [20]. Identified metabolites, formed during the degradation of MIT by *P. chrysosporium* were mono- and dihydroxylated methylisothiazolinon and N-methylmalonamic acid. The presence of hydroxylated derivatives indicates the involvement of hydroxylating enzymes in the biotransformation process. However, measurements of the activity of laccase, manganese peroxidase, lignin peroxidase, and cytochrome P450 did not confirm the involvement of these enzymes. It is noteworthy that the resulting MIT derivatives are less toxic than the parent compound against *D. magna* [20]. The process of the biodegradation of MIT by three strains of filamentous fungi, *Trichoderma longibrachiatum* FB01, *Aspergillus niger* FB14, and *Fusarium solani* FB07, occurs differently. Short-chain organic acids, such as tartaric acid, 2-oxobutanoic acid and acetic acid (*T. longibrachiatum*), malonic acid, 2-oxobutanoic acid, lactic acid, metoxiacetic acid, acetic acid (*A. niger*) and malonic acid, 2-oxobutanoic acid, propanoic acid, and acetic acid (*F. solani*) have been identified as metabolites of this preservative. The tested fungi were able to eliminate MIT in 16 h [154]. In both studies, stimulation of the growth of the tested micro-organisms was observed, which probably use this compound as a source of carbon and energy. So far, another pathway for the biodegradation of MIT by the microalgae *Scendesmus sp*. LX1 has been described. The algae completely removed MIT over 4 days and led to the cleavage of the ring by methylation and carboxylation [155].

### 4.4. Benzalkonium Chloride

Several reports have already described biologically mediated BAC degradation under laboratory conditions. The first report demonstrated the decomposition of BAC by 20 strains of *Burkholderia cepacia* bacteria. After an incubation period of 7 days, about 42.6% of BAC was eliminated. Benzyldimethylamine and benzylmethylamine were reported to be the metabolites present at the initial step of BAC degradation as a result of the cleavage of the C–alkyl-N bond. The authors identified two enzymes potentially responsible for C–N bond cleavage: amine oxidase and Rieske-type oxygenase. Moreover, eight catabolic enzymes involved in benzyldimethylamine degradation were identified and the complete degradation of the alkyl group of BAC was noted [156]. The isolation of BAC-degrading micro-organisms from a wide range of ecosystems has been described. Ertekin et al. isolated a strain highly resistant to BAC at a minimal inhibitory concentration of 1024 mg L^−1^ [157]. The identified *Pseudomonas sp*. BIOMIG1 was able to eliminate BAC within 3 days by leading to complete mineralization. The evaluation of immobilization as a better method for BAC elimination was described by Bergero et al. [158]. The comparison of BAC biodegradation by planktonic cells of *Aeromonas hydrophila* MFB03 isolated from industrial WWTPs to its degradation by Ca-alginate-encapsulated cells showed that immobilization increased the efficiency of elimination and, after 48 h, led to the utilization of 90% BAC as a sole carbon and energy source. Due to physical protection, immobilized cells are more resistant to BAC than free cells. Similar results were obtained for *Pseudomonas putida* ATCC 12633 [159]. Moreover, the use of a microbial consortium formed by these two strains and encapsulated in Ca-alginate is the most efficient method for BAC removal [158]. N,N-dimethylbenzylamine was observed as the result of the C–alkyl-N bond cleavage of BAC-C16 by two isolates from marine sediments, *Bacillus niabensis* and *Thalassospira sp*. These bacteria were able to degrade up to 90% BAC over 7 days [160]. Oh et al. studied the biodegradation of BAC as a sole carbon and energy substrate using a microbial community stemming from estuarine sediment and a member of the genus *Pseudomonas* [161]. Within 12 h, 80% of BAC degradation was observed in a bioreactor inoculated with mixed cultures without the detection of biotransformation products. In order to obtain energy, *P. nitroreducens,* with the use of amine oxidases, causes the dealkylation of BAC and the formation of two aldehyde products: dodecanal and tetradecanal aldehydes. The obtained metabolites are characterized by a lower toxicity than the parent compound. The aerobic hydroxylation of BAC catalyzed by mono-oxygenase possibly occurs in an enriched community of *Pseudomonas spp*. The cleavage of the C–alkyl-N bond leads to the formation of benzyldimethylamine. The authors suggested that benzyldimethylamine could be biotransformed by debenzylation to benzoic acid and dimethylamine. These transformations lead to a reduction in acute toxicity (Microtox) [162]. The algal degradation of BAC via pure cultures has been explored in seawater microalgae, *Tetrasemis suecica*. The tested organisms were able to successfully eliminate BAC-C12 and BAC-C14 from seawater and produced water, with rates of about 100% and 54% within 14 days of incubation, respectively. Furthermore, twelve isomeric intermediates, which are characterized by a lower tendency to be adsorbed into sediments than the parent compounds, were found. The authors suggested that the chemical reactions involved in the biodegradation pathways were multiple hydroxylations followed by dehydration. Hydroxylated BAC-C12 and dihydroxylated BAC-C14 were the most intense by-products formed during BAC-C12 and BAC-C14 transformation, respectively [163].

**Table 4:** Microbial degradation of cosmetic preservatives.

Compound	Micro-organism Used	Initial Concentration of Preservative	Removal [%]	Time Taken	Metabolites	References
TCC	Microbial consortium	975.4 µg kg^−1^	83.1	16 d	not analyzed	[143]
	*Pseudomonas fluorescens* MC46 (immobilized cells)	10 mg L^−1^	70.14–79.18	24 h	3,4-dichloroaniline; 4-chloroaniline; aniline; catechol	[144]
	*Pseudomonas fluorescens* MC46 (free cells)	10 mg L^−1^	42.12	24 h	3,4-dichloroaniline; 4-chloroaniline; aniline; catechol	[144]
	*Pseudomonas fluorescens* MC46 (immobilized cells)	5 mg L^−1^ 10 mg L^−1^ 20 mg L^−1^ 30 mg L^−1^ 40 mg L^−1^ 50 mg L^−1^	73.97 ± 0.03 78.26 ± 0.14 50.98 ± 0.27 27.05 ± 0.71 10.54 ± 0.10 7.88 ± 0.66	8 h 8 h 8 h 8 h 8 h 8 h	Not analyzed 3,4-dichloroaniline; 4-chloroaniline; aniline; Not analyzed Not analyzed Not analyzed Not analyzed	[145]
	*Pseudomonas fluorescens* MC46 (free cells)	5 mg L^−1^ 10 mg L^−1^ 20 mg L^−1^ 30 mg L^−1^ 40 mg L^−1^ 50 mg L^−1^	54.52 ± 0.06 44.73 ± 0.20 22.45 ± 0.27 16.98 ± 0.13 6.74 ± 0.01 4.30 ± 0.02	8 h 8 h 8 h 8 h 8 h 8 h	Not analyzed 3,4-dichloroaniline; 4-chloroaniline; aniline; Not analyzed Not analyzed Not analyzed Not analyzed	[145]
	*Pseudomonas fluorescens* MC46	9. 5 mg L^−1^	67 ± 2	6 h	Not analyzed	[146]
	*Sphingomonas sp.* YL-JM2C	4 mg L^−1^	35	5 d	3,4-dichloroaniline; 4-chloroaniline; 4-chlorocatechol;	[147]
	*Ochrobactrum sp.* TCC-2	5 mg L^−1^	56.70 ± 1.50	48 h	Not analyzed	[151]
	*Ochrobactrum sp.* MC22 (aerobic conditions)	9.40 mg L^−1^	78 ± 4.9	6 d	3,4-dichloroaniline; 4-chloroaniline;	[148]
	*Ochrobactrum sp.* MC22 (anaerobic conditions)	9.40 mg L^−1^	50%	14 d	3,4-dichloroaniline; 4-chloroaniline; aniline;	[148]
	*Ochrobactrum sp.* TCC-2 (aerobic conditions)	31.7 μM	96.88 ± 0.05	24 h	4-chloroaniline; 3,4-Dichloroaniline;	[149]
	*Ochrobactrum sp.* TCC-2 (anaerobic conditions)	31.7 μM	72.70 ± 2.90	24 h	4-chloroaniline; 3,4-Dichloroaniline;	[149]
PCMX	*Cunninghamella elegans* IM 1785/21GP	25 mg L^−1^	70	120 h	2,6-dimethylbenzene-1,4-diol, di-TMS; 2,5-dihydroxy-3-methylbenzaldehyde, di-TMS;	[124]
	*Trametes versicolor* IM 373	25 mg L^−1^	79	120 h	4,6-dioxohex-2-enoic acid, TMS; 5-methyl-6-oxohexa-2,4-dienoic acid, TMS; 3-chloro-2,4-dimethylhexa-2,4-dienedioic acid, di-TMS;	[124]
	*Aspergillus niger*	2 mg L^−1^	99	7 d	Not analyzed	[152]
	*Klebsiella pneumoniae* D2 (free cells)	8 mg L^−1^	55.7	24 h	Not analyzed	[164]
	*Klebsiella pneumoniae* D2 (immobilized cells)	8 mg L^−1^	88.3	24 h	Not analyzed	[164]
	Activated sludge	0.5 mg L^−1^	39.4 ± 17.3	72 h	Not analyzed	[153]
	Activated sludge	5 mg L^−1^	49.4 ± 15	72 h	Not analyzed	[153]
MIT	*Pchanerochaete chrysosporium*	50 µg L^−1^ and 30 mg L^−1^	100	12 h	monohydroxylated MIT; dihydroxylated MIT; N-methylmalonamic acid;	[20]
	*Trichoderma longibrachiatum* FB01	10 g L^−1^	100	16 h	tartaric acid; 2-oxobutanoic acid; acetic acid;	[154]
	*Aspergillus niger* FB14	10 g L^−1^	100	16 h	malonic acid; 2-oxobutanoic acid; lactic acid; metoxiacetic acid; acetic acid;	[154]
	*Fusarium solani* FB07	10 g L^−1^	100	16 h	malonic acid; 2-oxobutanoic acid; propanoic acid; acetic acid;	[154]
BAC	20 strains of *Burkholderia cepacia*	34–64 mg L^−1^	4.7 ± 2.4–42.6 ± 12.3	7 d	benzyldimethylamine; benzylmethylamine	[156]
	*Pseudomonas sp*. BIOMIG1	200 µM	62.5	3 d	mineralization	[157]
	*Aeromonas hydrophila* MFB03 (immobilized cells)	25–210 mg L^−1^	90	48 h	Not analyzed	[158]
	*Aeromonas hydrophila* MFB03 (free cells)	50 mg L^−1^	74.2 ± 2.3–80.4 ± 0.6	48 h	Not analyzed	[158]
	*Pseudomonas putida* ATCC 12633 (immobilized cells)	50 mg L^−1^	74 ± 4.70	48 h	Not analyzed	[158]
	*Pseudomonas putida* ATCC 12633 (immobilized cells)	105–315 mg L^−1^	90	24 h	Not analyzed	[159]
	*Bacillus niabensis*	2 mg mL^−1^	Up to 90	7 d	N,N-dimethylbenzylamine	[160]
	*Thalassospira sp*	4 mg mL^−1^	Up to 90	7 d	N,N-dimethylbenzylamine	[160]
	Microbial community	50 mg L^−1^	80	12 h	Not detected	[161]
	Microbial community	50 mg L^−1^	100	24 h	benzyldimethylamine	[162]
	*Tetrasemis suecica*	5 mg L^−1^	100	3–6 d	OH-BAC-C12; 2OH-BAC-C14	[163]

## 5. Conclusions

This review demonstrates that, due to their broad application in many products and no effective removal from wastewater treatment plants, triclocarban, chloroxylenol, methylisothiazolinone, and benzalkonium chloride are often detected as emerging contaminants in sewage as well as in receiving environments, including surface water, sediments, and soils. Dischargers from WWTPs were identified as the major source of these xenobiotics in the natural environment, although biosolid-amended soils and reclaimed water for irrigation may also be important sources of preservatives in the environment. The concentrations of biocide residues ranged from ng L^−1^ to ug L^−1^ in WWTPs and surface waters and from ng g^−1^ to ug g^−1^ in sediments and soils. Among all discussed preservatives, TCC and BAC were the most frequently described. The factors influencing their occurrence include population size, consumption, social level, seasons, and wastewater treatment technology. The occurrence and prevalence of the discussed micropollutants in the environment are of increasing interest, owing to their toxicity potential on non-target organisms, such as aquatic and terrestrial ones. The disadvantageous effects include neurotoxicity, genotoxicity, embryotoxicity, growth inhibition, abnormality in motility, lifespan, hatching, and endocrine-disrupting effects. Several micro-organisms, such as strains of bacteria, fungi, microalgae as pure and mixed cultures, and free or immobilized cells, were found to be capable of degrading preservatives. With the availability of sensitive chromatographic and mass spectrometric methods, it was possible to identify and characterize formed metabolites. A decrease or increase in the toxicity of emerging products compared to parent compounds was also noted.

Here, we recommend the following key areas for research:

- Because of the market development, extensive use, and continuous discharge of personal care products, there is a need for more detailed data on the environmental occurrence, mainly for chloroxylenol and methylisothiazolinone.
- Toxicological studies on the chronic effects of the environmental concentrations of single preservatives and their metabolites should be considered, as well as the effects of a mixture of pollutants on aquatic and soil organisms.
- Mechanisms of the microbial biodegradation of preservatives and their metabolites should also be better understood, which will make it possible to design a treatment technology that is both effective and affordable for limiting the release of pollutants.

## Figures and Tables

**Table 1:** Basic information on the target preservatives.

INCI Name	Triclocarban	Chloroxylenol	Methylisothiazolinone	Benzalkonium Chloride
Acronym	TCC	PCMX	MIT	BAC
CAS Number	101-20-2	88-04-0/1321-23-9	2682-20-4	63449-41-2/68391-01-5/68424-85-1/85409-22-9
Formula	C_13_H_9_Cl_3_N_2_O	C_8_H_9_OCl	C_4_H_5_NOS	C_9_H_13_ClNR (R = C_8_H_17_ to C_18_H_37_)
Molecular weight	315.58 g mol^−1^	156.61 g mol^−1^	115.1 g mol^−1^	-
Structure	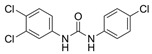	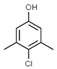	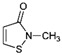	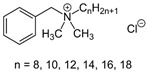

## Data Availability

Not applicable.
